# Association between stressful life events and resting heart rate

**DOI:** 10.1186/s40359-014-0029-0

**Published:** 2014-09-08

**Authors:** Ju-Mi Lee, Hyeon Chang Kim, Jee In Kang, Il Suh

**Affiliations:** Department of Preventive Medicine, Yonsei University College of Medicine, 50 Yonsei-ro, Seodaemun-gu, Seoul 120-752 Republic of Korea; Department of Preventive Medicine, Northwestern University Feinberg School of Medicine, Chicago, IL USA; Department of Psychiatry, Yonsei University College of Medicine, Seoul, South Korea

**Keywords:** Life change events, Heart rate, Psychological stress

## Abstract

**Background:**

Despite a diverse literature, the association between stress and various cardiovascular conditions remains controversial. Moreover, a direct association between stressful life events (SLEs) and heart rate (HR) have not been fully investigated. This study evaluated the association between SLEs and resting HR in middle-aged Koreans.

**Methods:**

A cross-sectional analysis was conducted for 1,703 men and 2,730 women aged 27–87 years from the community-based Korean Genome and Epidemiology Study-Kanghwa study. All participants completed a baseline health examination. The life experience survey questionnaire was administered to measure SLEs experienced during the past 3 months. Resting blood pressure and HR were measured twice over a 5 minute interval. If the difference in blood pressure was more than 10 mmHg, then a third blood pressure and HR measurement was taken after 5 minutes of rest. The average of the last two measurements was used for analysis. The association between SLEs and HR was assessed by correlation and multiple linear regression analysis.

**Results:**

Compared with people with no SLEs (mean HR of 67.30 beats/min), HR was significantly lower in those who experienced one (mean HR of 65.64 beats/min, p = 0.002), two (mean HR of 63.73 beats/min, p < 0.001), and 3+ SLEs (mean HR of 64.17 beats/min, p < 0.001). This association was observed even after adjustment for sex, age, body mass index, hypertension treatment, oral contraceptive use, postmenopausal hormone therapy, thyroid disease, liver disease, cigarette smoking use, alcohol drinking use, and blood urea nitrogen to creatinine ratio. Compared with people with no SLEs, those with 1, 2, and 3+ SLEs had a lower resting HR by 1.485 (p = 0.005), 3.718 (p < 0.001), and 3.176 (p < 0.001), respectively.

**Conclusion:**

Our findings suggest that the experience of a recent SLEs are associated with a lower resting HR in Korean adults. Although further investigation is required, people who have experienced recent SLEs and have a lower HR than usual may need attention for their stress level.

## Background

Heart rate (HR) is the most frequently and noninvasively measured vital sign in diverse settings. People can easily have their HR checked alone or along with blood pressure at hospitals, clinics, community health centers, and fitness centers. The overall mean HR in the United States population is 71 beats/min for adult males and 74 beats/min for adult females (Ostchega et al. 2011). A higher resting HR is associated with future cardiovascular conditions such as cardiovascular mortality (Kannel et al. 1987; Dyer et al. 1980; Greenland et al. 1999).

Following the discovery of a general physiological response to stress, the concept of stress as a risk factor for cardiovascular conditions has been suggested. Previous studies have reported that stress is associated with various cardiovascular conditions such as cardiovascular disease (Iso et al. 2002; Mostofsky et al. 2012; Engstrom et al. 2004), metabolic syndrome (Fabre et al. 2013; Raikkonen et al. 2007; Pyykkonen et al. 2010), higher and lower blood pressure (Melamed et al. 1997; Fallo et al. 2002), and decreased HR variability (Lucini et al. 2005; Bernatova et al. 2002). One approach to measure stress is using life events, which can be used to quantify how environmental stressors affect health (Rabkin and Struening 1976). “In general, the purpose of life events research is to demonstrate a temporal association between the onset of illness and a recent increase in the number of events that require socially adaptive responses on the part of individual (Rabkin and Struening 1976).” (Meyer 1951) has suggested the use of a life chart for the diagnosis of clinical disease (Haney 1980). Selye (Haney 1980) was the first to suggest that both positive and negative perceptions of stress play important roles in the etiology of disease. Kissen (Haney 1980) was one of the first investigators to study the association between illness and life events. Many studies have been conducted to assess the association between life events and cardiovascular diseases (Haney 1980). However, the results of such studies remain controversial. Based on the results from general descriptive studies, Rahe (Rahe and Lind 1971; Haney 1980) reported that life events were found to be significantly higher for the diseased subject in the year before their death as compared with the period two years before their death. Other studies, however, have failed to detect a difference in the mean level of life changes in the 6 months prior to a heart attack (Rahe and Paasikivi 1971). Based on the results from analytic studies, Lundberg reported that individual appraisals about life events have a significant impact on heart disease (Lundberg et al. 1975; Haney 1980). This result supports Lazarus’s opinion that individual appraisals of life events are important.

**Figure 1:**
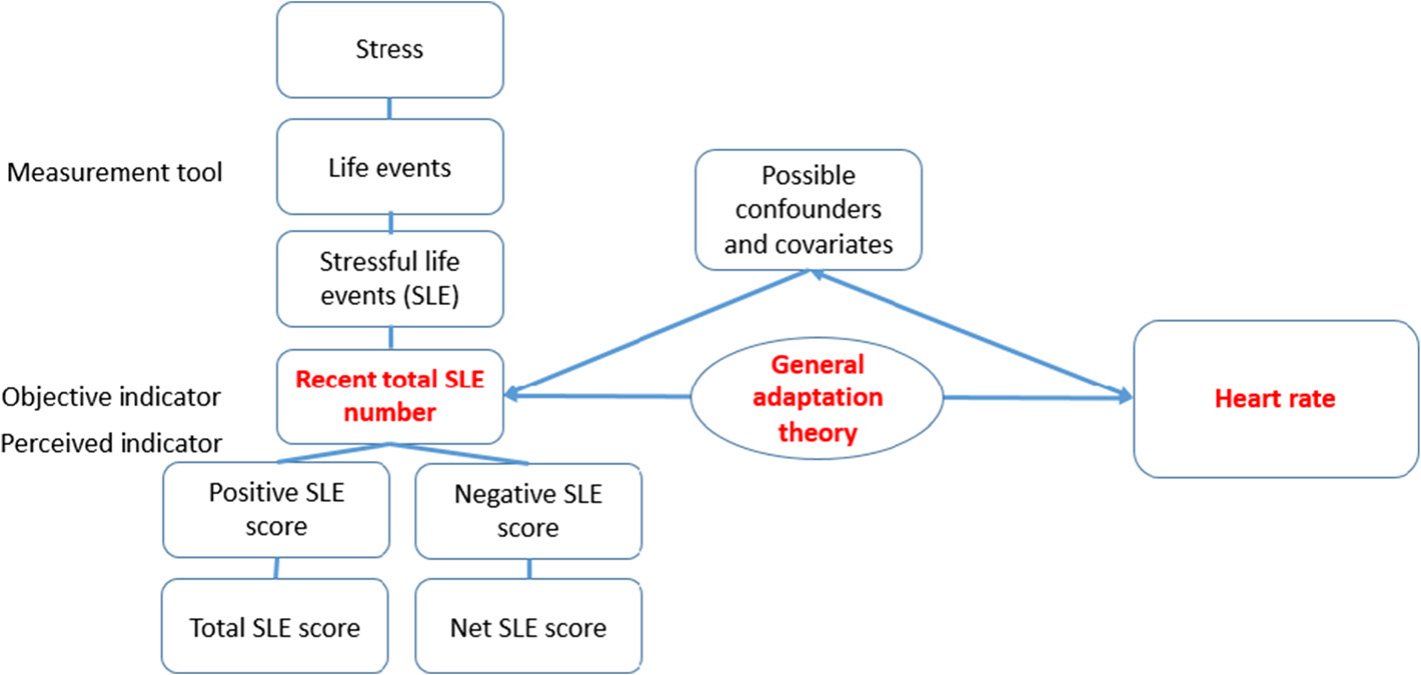
**Study framework.**

Despite the existence of a diverse literature, the association between stress and various cardiovascular conditions remains controversial. Moreover, the direct association between a stressful life events (SLEs) and HR have not been fully investigated. This study aimed to evaluate the association between SLEs and resting HR in community-dwelling middle-aged adults in Korea. Our main hypothesis was that the more total number of SLEs exist, the higher HR present. We considered Selye’s concept of stress and general adaptation syndrome as the fundamental framework and both negative and positive SLEs are influence on adaptation; therefore, stages of resistance and exhaustion are expected to play a role in this associations (Figure 1). This study is distinct from other studies in that it 1) focuses on the direct association between SLEs and HR (intermediate outcome) rather than cardiovascular disease, which to our knowledge is the first study to explore this association; 2) participants were taken from a general community-dwelling population rather than a patient population or population of workers from a specific occupation; 3) life events exposure were selected naturally due to the use of baseline cohort data; and 4) data were extracted from the baseline measurements of a cardiovascular cohort study, which means that all confounders and covariates were objectively measured.

## Methods

### Study participants

The study design was a cross-sectional analysis of baseline data collected for an ongoing community-based prospective cohort study. The Korean Genome and Epidemiologic Study (KoGES)-Kangwha study started in 2006 and has enrolled community dwellers from Kangwha Island, Incheon, South Korea. The KoGES-Kangwha cohort study was designed to examine cardiovascular diseases through the use of questionnaires, physical examinations, various types of biomarkers, and DNA analysis. Detailed methods for the KoGES-Kangwha study have been reported elsewhere (Lee et al. 2009). This analysis enrolled participants who completed baseline health examinations between 2006 and 2010. Among the 4,446 total participants, we excluded eight participants because their answers were inconsistent with the life experience survey questionnaire. An additional five participants were excluded for the following reasons: systolic blood pressure <60 mmHg or missing, diastolic blood pressure <40 mmHg or missing, and HR <30 beats/min or missing. A total of 4,433 participants (1,703 male and 2,730 female) aged 27–87 years were included in this analysis. All participants signed written informed consent. The study protocol was approved by the Institutional Review Board of Yonsei University Health System (4-2004-0105, 4-2006-0101, 4-2008-0135, 4-2009-0270, 4-2010-0272) and monitored by the Human Research Protection Center of Severance Hospital, Yonsei University Health System.

### Measurements

A trained research examiner obtained medical and family history, health behaviour, SLEs, and information about diet from all participants using standardized questionnaires. We measured SLEs and their effects using the Korean version of the life experience survey questionnaire (Lee 1993), which was originally developed and validated by Sarason, Johnson, and Siegel (Sarason et al. 1978) and lists 57 possible life events such as marriage, bereavement, major change in lifestyle, and educational and occupational successes and failures. Additionally, participants were asked to indicate any important event not included in the list. We limited our analysis to SLEs that occurred within the past three months to minimize recall bias (Dohrenwend 2006; Raphael et al. 1991). The total number of SLEs was used as a relatively objective measure of stress. The positive or negative effect of each life event was classified by the participant on a seven-point scale to measure perceived stress. Extremely negative, moderately negative, slightly negative, neither negative nor positive, slightly positive, moderately positive, and extremely positive influences were coded as −3, −2, −1, 0, +1, +2, and +3 points, respectively (Sarason et al. 1978). We calculated five stress indices (Sarason et al. 1978) from the life experience survey questionnaire: the total number of SLEs, the total SLE score (the sum of the absolute value of all SLE points), the negative SLE score (the sum of the absolute value of the negative SLE points), the positive SLE score (the sum of the positive SLE points), and the net SLE score (the absolute value of the total negative SLE score minus the total positive SLE score). All participants completed the health examination, including blood pressure measurements, blood laboratory tests, and electrocardiography, during one research clinic visit. Participants were required to refrain from smoking or ingesting caffeine for eight hours prior to the health examination. Participants were asked to sit and rest in a room for at least five minutes prior to the HR and blood pressure measurements. With participants seated, an appropriate-sized cuff was applied snugly around the upper right arm at the level of the heart. The appropriate cuff size was chosen for each subject according to their mid-arm circumference. We took the first blood pressure measurement, waited for 5 minutes, and then took the second blood pressure measurement using an automatic sphygmomanometer (Dinamap 1846 SX/P; GE Healthcare, USA). If the difference between the first and the second blood pressure was more than 10 mmHg, then a third blood pressure measurement was taken 5 minutes later (among 4,433 participants, 102 males and 226 females underwent a third blood pressure measurement). The mean value of the last 2 measurements was computed from the blood pressure and HR measurements. Bradycardia was defined as a resting HR less than 60 beats/min. A standard 12-lead electrocardiogram with a recording speed of 25 mm/sec (Page Writer Trim III M1707A, Philips, Netherlands) was conducted in all participants. Electrocardiogram recordings were conducted in a quiet, isolated room and subjects were maintained in the supine position. The HR and QT intervals were analyzed automatically by the Philips software program12-lead Algorithm (Philips, Netherlands), and electrocardiogram findings were confirmed by a cardiologist.

Fasting blood samples were collected from the antecubital vein following at least eight hours of fasting. Blood samples were sent to a centralized research laboratory for analysis. Blood urea nitrogen (BUN) and creatinine (Cr) were measured by colorimetric methods using automatic analyzers (ADVIA 1650, Bayer Corp, USA). The total body water content was estimated through the use of a tetrapolar lead system bio-impedance (BIA Fatness Analyzer GIF-891DX, Gilwoo Trading Co, Korea). Standing height was measured to the nearest 0.1 cm on a stadiometer (SECA763, SECA GMBH, Germany). Participants removed hair ornaments from the tops of their heads to measure stature properly, and stood on the floor with the heels of both feet together and toes pointed slightly outward at an approximately 60° angle. The position of the heels, buttocks, shoulder blades, and back of the head was checked for contact with the vertical backboard. Once participants were correctly positioned, they took a deep breath and the headboard was lowered and positioned firmly on top of the head with sufficient pressure to compress the hair, and height was recorded by the examiners. At the same time body weight was measured to the nearest 0.1 kg on a digital scale with participants wearing underwear and examination gowns only. Weight was recorded by the examiners when the digital readout was stable. Body mass index (BMI) was calculated as the weight (kg) divided by the height (m^2^). We used questionnaires to assess cigarette smoking and alcohol drinking use.

### Statistical analysis

All participants were classified by the presence of recent SLEs, and those with SLEs were further classified into three groups according to the total number of SLEs (i.e., one, two, and three or more SLEs, respectively). Characteristics were compared between groups, and their differences were tested by Chi-square test or ANOVA as appropriate. Correlations between stress indices and other variables were assessed by Pearson’s correlation coefficients. Multiple linear regression analysis was conducted to evaluate the association between SLEs and resting HR. We calculated the unadjusted and adjusted ß coefficients and *p* values using the participants without SLEs as the reference group. In the adjusted model, we controlled for age, BMI, hypertension treatment status, oral contraceptive use, postmenopausal hormone therapy, history of thyroid disease, history of liver disease, cigarette smoking use (never, former, or current), alcohol drinking use (never, former, or current), and BUN to Cr ratio. Age, BMI, hypertension treatment status, oral contraceptive use, postmenopausal hormone therapy, history of thyroid disease, and history of liver disease were selected based on the existing literature. Lifestyle factors such as smoking status and alcohol drinking use were selected because they may influence both SLEs and HR. The BUN to Cr ratio was additionally added as a marker of kidney function, which may alter the circulation volume and therefore change HR. All of these variables are associated with HR. All statistical analyses were performed using SAS version 9.2.0 (SAS Institute, Cary, NC, USA). A p*-*value <0.05 was considered to be statistically significant using a two-tailed test.

## Results

The five most frequent SLEs were: major change in sleeping habits (n = 183), major change in eating habits (n = 158), serious illness or injury of a close family member (n = 129), death of a close family member (n = 115), and trouble with in-laws and relatives (n = 89).

The characteristics of the study participants are summarized according to the total number of SLEs separately for males and females (Table 1 and 2, respectively). Age (M: p < 0.001, F: p < 0.001), resting HR (M: p < 0.001, F: p < 0.001), the QRS duration on the electrocardiogram (M: p = 0.003, F: p < 0.001), the prevalence of bradycardia (M: p = 0.051, F: p < 0.001), and the total body water content (M: p = 0.030, F: p < 0.001) varied by the total number of SLEs in both sexes. The total number of SLEs was significantly associated with cigarette smoking use (p = 0.006) in males only, whereas it was associated with systolic blood pressure (p = 0.020), the PR interval on the electrocardiogram (p = 0.038), the BUN to Cr ratio (p = 0.017), hematocrit (p < 0.001), and alcohol drinking use (p = 0.006) in females only. Men with a higher number of SLEs tended to report less cigarette use. Women with a higher number of SLEs tended to have longer PR intervals on the electrocardiogram, a higher BUN to Cr ratio, and higher alcohol use.

**Table 1:** **Participant characteristics depending on the total number of SLEs among 1,703 males**

**Variables**	**Total number of SLEs**				**p**
	**0 (n = 1,418)**	**1 (n = 142)**	**2 (n = 64)**	**≥3 (n = 79)**	
Age, years	57.1 ± 8.8	60.6 ± 9.8	59.8 ± 9.1	56.9 ± 8.9	<0.001^a^
Body mass index, kg/m^2^	24.3 ± 2.9	24.4 ± 3.0	23.9 ± 2.8	24.1 ± 3.2	0.701^a^
Systolic BP, mmHg	122.0 ± 17.1	123.3 ± 16.2	120.0 ± 16.6	122.8 ± 16.4	0.616^a^
Diastolic BP, mmHg	77.7 ± 10.0	77.0 ± 9.6	76.5 ± 10.8	78.0 ± 10.7	0.687^a^
Heart rate, bpm	66.3 ± 10.5	64.4 ± 9.5	61.8 ± 7.9	64.2 ± 10.2	<0.001^a^
Heart rate on ECG, bpm	62.5 ± 10.3	60.2 ± 8.4	59.1 ± 8.3	61.0 ± 9.5	0.003^a^
PR interval on ECG, ms	172.9 ± 23.2	174.9 ± 21.6	174.8 ± 28.5	172.0 ± 23.8	0.700^a^
QRS duration on ECG, ms	94.5 ± 12.5	91.3 ± 19.0	92.5 ± 23.7	90.0 ± 17.4	0.005^a^
BUN to Cr ratio	15.4 ± 4.1	15.8 ± 4.4	15.5 ± 3.9	15.7 ± 4.7	0.558^a^
Hematocrit, %	44.4 ± 3.4	44.8 ± 4.3	44.2 ± 4.0	44.6 ± 3.8	0.532^a^
Total body water, kg	39.0 ± 6.4	40.6 ± 7.8	39.9 ± 6.9	39.4 ± 6.6	0.030^a^
Bradycardia	391 (27.6)	51 (35.9)	23 (35.9)	28 (35.4)	0.051^b^
Thyroid disease history	17 (1.2)	0 (0.0)	0 (0.0)	1 (1.3)	0.474^c^
Liver disease history	5 (0.4)	0 (0.0)	0 (0.0)	1 (1.3)	0.456^c^
Hypertension	498 (35.1)	62 (43.7)	23 (35.9)	30 (38.0)	0.238^b^
Hypertension treatment	325 (22.9)	43 (30.3)	16 (25.0)	16 (20.3)	0.217^b^
Regular exercise status	517 (36.5)	49 (34.5)	20 (31.3)	31 (39.2)	0.750^b^
Cigarette smoking					
Never	452 (31.9)	36 (25.4)	22 (34.4)	29 (36.7)	0.006^b^
Former	508 (35.8)	73 (51.4)	28 (43.8)	31 (39.2)	
Current	458 (32.3)	33 (23.2)	14 (21.9)	19 (24.1)	
Alcohol drinking					
Never	359 (25.3)	41 (28.9)	22 (34.4)	23 (29.1)	0.345^b^
Former	163 (11.5)	22 (15.5)	8 (12.5)	8 (10.1)	
Current	896 (63.2)	79 (55.6)	34 (53.1)	48 (60.8)	

Data are expressed as the mean ± SD or the number (%).

^a^p-value was calculated by an ANOVA between groups.

^b^p-value was calculated by a Chi-square test between groups.

^c^p-value was calculated by a Fisher’s exact test between groups.

**Table 2:** **Participant characteristics depending on the total number of SLEs among 2,730 females**

**Variables**	**Total number of SLEs**				**p**
	**0 (n = 2,220)**	**1 (n = 260)**	**2 (n = 107)**	**≥3 (n = 143)**	
Age, years	55.7 ± 8.6	58.6 ± 10.2	59.1 ± 9.6	54.8 ± 8.9	<0.001^a^
Body mass index, kg/m^2^	24.8 ± 3.3	24.8 ± 3.6	25.2 ± 3.2	25.0 ± 2.8	0.540^a^
Systolic BP, mmHg	119.6 ± 18.2	122.9 ± 19.0	122.4 ± 18.5	118.8 ± 17.5	0.020^a^
Diastolic BP, mmHg	72.2 ± 10.2	71.7 ± 10.2	71.3 ± 9.3	71.3 ± 10.0	0.539^a^
Heart rate, bpm	68.0 ± 9.6	66.3 ± 10.4	64.9 ± 9.6	64.2 ± 8.9	<0.001^a^
Heart rate on ECG, bpm	64.3 ± 9.1	62.8 ± 9.3	61.7 ± 8.7	62.1 ± 11.3	<0.001^a^
PR interval on ECG, ms	164.4 ± 21.0	166.8 ± 21.0	169.3 ± 25.5	166.3 ± 21.1	0.038^a^
QRS duration on ECG, ms	87.5 ± 14.7	85.6 ± 19.9	84.7 ± 23.6	82.6 ± 18.5	0.005^a^
BUN to Cr ratio	17.2 ± 4.9	18.2 ± 5.1	17.3 ± 4.5	17.4 ± 4.6	0.017^a^
Hematocrit, %	39.3 ± 3.0	40.0 ± 2.9	39.8 ± 3.2	39.1 ± 3.2	<0.001^a^
Total body water, kg	29.7 ± 4.5	30.3 ± 5.6	31.0 ± 6.5	31.4 ± 6.02	<0.001^a^
Bradycardia	406 (18.3)	59 (22.7)	29 (27.1)	44 (30.8)	<0.001^b^
Thyroid disease history	98 (4.4)	14 (5.4)	7 (6.5)	4 (2.8)	0.473^c^
Liver disease history	10 (0.5)	0 (0.0)	0 (0.0)	1 (0.7)	0.589^c^
Hypertension	753 (33.9)	100 (38.5)	41 (38.3)	52 (36.4)	0.390^b^
Hypertension treatment	524 (23.6)	72 (27.7)	29 (27.1)	42 (29.4)	0.201^b^
Oral contraceptive use	6 (0.3)	1 (0.4)	0 (0.0)	1 (0.7)	0.744^c^
Hormone therapy	117 (5.3)	6 (2.3)	2 (1.9)	7 (4.9)	0.087^c^
Regular exercise status	724 (32.6)	95 (36.5)	34 (31.8)	53 (37.1)	0.438^b^
Cigarette smoking					
Never	2,134 (96.1)	252 (96.9)	104 (97.2)	139 (97.2)	0.976^c^
Former	35 (1.6)	3 (1.2)	1 (0.9)	2 (1.4)	
Current	51 (2.3)	5 (1.9)	2 (1.9)	2 (1.4)	
Alcohol drinking					
Never	1,560 (70.3)	185 (71.2)	79 (73.8)	88 (61.5)	0.006^c^
Former	70 (3.2)	18 (6.9)	3 (2.8)	9 (6.3)	
Current	590 (26.6)	57 (21.9)	25 (23.4)	46 (32.2)	

Data are expressed as the mean ± SD or the number (%).

^a^p-value was calculated by an ANOVA between groups.

^b^p-value was calculated by a Chi-square test between groups.

^c^p-value was calculated by a Fisher’s exact test between groups.

The total number of SLEs was positively correlated with age (Table 3). The total number of SLEs was inversely associated with resting HR (M: r = −0.084, p < 0.001; F: r = −0.111, p < 0.001), the HR on the electrocardiogram (M: r = −0.079, p = 0.001; F: r = −0.077, p < 0.001), and the QRS duration on the electrocardiogram (M: r = −0.085, p = 0.001; F: r = −0.073, p < 0.001) in both sexes. Pulse pressure (r = 0.049, p = 0.011), the PR interval on the electrocardiogram (r = 0.044, p = 0.022), and the total body water content (r = 0.093, p < 0.001) were positively correlated with the total number of SLEs in females only. The total number of SLEs was highly correlated with other SLE indices such as the total SLE score (r = 0.769, p < 0.001), the negative SLE score (r = 0.650, p < 0.001), the positive SLE score (r = 0.589, p < 0.001), and the net SLE score (r = 0.165, p < 0.001), as expected.

**Table 3:** **Correlations between participant characteristics and total number of SLEs**

**Variables**	**Total (n = 4,433)**		**Male (n = 1,703)**		**Female (n = 2,730)**	
	**Pearson’s coefficient**	**p**	**Pearson’s coefficient**	**p**	**Pearson’s coefficient**	**p**
Age	**0.044**	**0.004**	**0.053**	**0.029**	**0.041**	**0.033**
Body mass index	0.010	0.507	−0.018	0.463	0.022	0.246
Systolic blood pressure	0.013	0.386	0.004	0.855	0.020	0.304
Diastolic blood pressure	−0.025	0.092	−0.009	0.698	−0.027	0.154
Pulse pressure	**0.039**	**0.009**	0.015	0.540	**0.049**	**0.011**
Heart rate	**−0.098**	**<.001**	**−0.084**	**<.001**	**−0.111**	**<.001**
Heart rate on ECG	**−0.076**	**<.001**	**−0.079**	**0.001**	**−0.077**	**<.001**
PR interval on ECG	0.026	0.090	0.008	0.754	**0.044**	**0.022**
QRS duration on ECG	**−0.075**	**<.001**	**−0.085**	**0.001**	**−0.073**	**<.001**
BUN to Cr ratio	**0.031**	**0.039**	0.026	0.294	0.029	0.132
Hematocrit	0.004	0.808	0.015	0.543	0.023	0.230
Total body water	**0.041**	**0.006**	0.043	0.081	**0.093**	**<.001**
Total SLE score	**0.769**	**<.001**	**0.815**	**<.001**	**0.756**	**<.001**
Negative SLE score	**0.650**	**<.001**	**0.715**	**<.001**	**0.633**	**<.001**
Positive SLE score	**0.589**	**<.001**	**0.579**	**<.001**	**0.594**	**<.001**
Net SLS score	**0.165**	**<.001**	**0.137**	**<.001**	**0.179**	**<.001**

p-value was calculated by a Pearson’s correlation test.

Table 4 shows the association between the total number of SLEs and resting HR over all participants and by sex. Participants with a recent SLE had a significantly lower resting HR compared with participants who did not have a recent SLE. Furthermore, there was a decrease in HR as the number of SLEs increased (Total β = −3.133, p < 0.001). This association was consistent before and after adjustments for age, BMI, hypertension treatment status, oral contraceptive use, postmenopausal hormone therapy, history of thyroid disease, history of liver disease, cigarette smoking use, alcohol drinking use, and BUN to Cr ratio (Total β = −3.176, p < 0.001). Moreover, this independent association was observed in both the pooled and sex-specific analyses.

**Table 4:** **Association between the total number of SLEs and resting HR**

**Total number of SLEs**	**Unadjusted**		Adjusted*	
	**β coefficient**	**p**	**β coefficient**	**p**
Total (n = 4,433)				
0 (n = 3,638)	Reference	–	Reference	–
1 (n = 402)	−1.655	0.002	−1.485	0.005
2 (n = 171)	−3.568	<0.001	−3.718	<0.001
≥3 (n = 222)	−3.133	<0.001	−3.176	<0.001
Male (n = 1,703)				
0 (n = 1,418)	Reference	–	Reference	–
1 (n = 142)	−1.820	0.045	−1.790	0.050
2 (n = 64)	−4.483	<0.001	−4.469	<0.001
≥3 (n = 79)	−2.066	0.084	−2.009	0.092
Female (n = 2,730)				
0 (n = 2,220)	Reference	–	Reference	–
1 (n = 260)	−1.662	0.009	−1.395	0.028
2 (n = 107)	−3.063	0.001	−3.278	<0.001
≥3 (n = 143)	−3.812	<0.001	−3.742	<0.001

p-value was calculated by a multiple linear regression analysis.

*Adjusted for age, body mass index, hypertension treatment, oral contraceptive use (in females), postmenopausal hormone therapy (in females), history of thyroid disease, history of liver disease, cigarette smoking, alcohol drinking, and BUN to Cr ratio.

## Discussion

Contrary to our hypothesis, people with recent SLEs had a lower resting HR than those who did not have recent SLEs in middle aged Koreans. Moreover, an increase in the number of SLEs was consistently associated with a decrease in resting HR, even after adjusting for potentially confounding variables.

The importance of our study is the first to examine the associations between recent SLEs and resting HR in a community-based Korean population as far as we know. Only a few studies have directly assessed the association between emotional stress and resting HR. Many studies have reported that emotional stress or stressful events are associated with HR variability (Lucini et al. 2005; Bernatova et al. 2002; Mezzacappa et al. 2001; Schmidt et al. 2010), cardiac reactivity (Ginty and Conklin 2011), and cardiac arrhythmia (Lynch et al. 1977). Our study can be distinguished from these other studies in that the study variable we examined, resting HR, is simple to measure and readily available from any population. Many previous studies have investigated the effects of stress in animal models (Bernatova et al. 2002; Schmidt et al. 2010; Sarenac et al. 2011) or in special populations such as posttraumatic disorder patients (Sahar et al. 2001), shift workers (Furlan et al. 2000), or chronic psychosocial stress patients (Lucini et al. 2005). Studies among relatively healthy community-dwelling individuals are limited. Many previous studies have assessed the short-term effects of stress by comparing outcome variables before and after stress interventions (Mezzacappa et al. 2001; Schmidt et al. 2010; Ginty and Conklin 2011; Sahar et al. 2001; Furlan et al. 2000). However, people are exposed to stress mainly through certain stressful events in their personal and occupational lives. For this reason, many observational studies use SLEs, which are measured by the life experience survey questionnaire, as an objective measure of stress. Our results provided careful insight that decreased HR in people who have experienced recent SLEs is less likely to be related to abnormal conduction or muscle weakness and more likely to be related to vagal rebound effects as observed in the RR interval and the QRS duration. Our data was collected in a well-established community-based cohort study using standardized methodology to ascertain SLEs and HR as well as confounding factors.

The association between recent SLEs and lower HR can be explained in several ways. First, vagal rebound effects following psychological stress may decrease resting HR (Mezzacappa et al. 2001). Hyperactivity of the vagus nerve during stressful situations (Alboni et al. 2011) and decreased HR following external stress (Lucini et al. 2005; Bernatova et al. 2002; Mezzacappa et al. 2001; Smeets 2010) have been reported previously. In our study, the QRS duration on the electrocardiogram decreased as the number of SLEs increased in both males and females. This result implies that both conduction from the atrioventricular node to the ventricles and ventricle contraction tend to be faster, although HR (as measured from the RR interval on the electrocardiogram) decreased as the total number of SLEs increased. These findings support that decreased HR in people who have experienced recent SLEs is less likely to be related to abnormal conduction or muscle weakness and is more likely to be related to a vagal rebound effect. Second, adaptation to stressful conditions may explain the decreased HR. Our study measured SLEs that occurred during the previous three months, which may overlap with the recovery stage of the psychological stress. During the recovery stage, the vagal rebound may be crucial in restoring cardiovascular homeostasis (Mezzacappa et al. 2001). In cardiopulmonary training studies, physical exercise raised HR in the short term but lowered resting HR over the long term following physical training (HJ 2000). It might be also possible that HR can be lowered following exposure to psychological stress. Third, the decreased HR may be due to an aldosterone effect. Emotional stress, pain, and surgical stress may influence aldosterone production directly via nervous control (Venning et al. 1957) and indirectly through the hypothalamic-pituitary-adrenal axis. Aldosterone causes fluid retention and increased plasma volume in the human body. Increased plasma volume means an increased preload of the heart, which can decrease HR. We did not measure aldosterone levels or plasma volume status, but we calculated total body water content from the bioelectrical impedance measurement. In our study, the number of SLEs was positively associated with the total body water content, but not associated with the BUN to Cr ratio. These findings suggest that the number of SLEs may be associated with plasma volume expansion without renal impairment.

Our study has several limitations. First, we could not assess the temporal relationship between SLEs and HR due to the cross-sectional study design. However, we asked about life events during the last three months and assessed their association with the current resting HR. Second, there is considerable intracategory variability in the life experience survey questionnaire (Dohrenwend 2006). The number of SLEs can be considered an objective indicator of stress when compared with measurements from the perceived stress questionnaire. However, life events listed in the questionnaire can be intracorrelated. For example, a person with a major personal illness may lose his/her job because of the disease and then have a financial crisis due to the job loss. The effect of similar life events may therefore vary considerably across individuals. We attempted to minimize this effect by limiting SLEs to events that occurred during the previous three months. Third, there is the possibility of residual confounding factors. We could not control for the effects of personal characteristics or susceptibility to stressful events. Similar life events may vary in the direction (positive or negative) and magnitude of their effects between individuals. We therefore assessed several indicators of perceived stress, including the total SLE score, the negative SLE score, the positive SLE score, and the net SLE score. We observed similar associations of these scores with resting HR, but there may be additional unmeasured confounders.

## Conclusion

In conclusion, our findings suggest that recent SLEs are associated with lower resting HR.

Measurements of HR and SLEs are relatively simple, inexpensive, and non-invasive and can be applied to almost any population. Unexpectedly, our findings suggest that recent SLEs may confer a beneficial effect to systemic circulation by lowering HR. This study suggests that future screening and stress level intervention efforts should target middle-aged individuals who may benefit the most from treatment, namely those with recent SLEs and lower HR. However, further investigation is required to examine the temporal relationship between SLEs and changes in HR to develop a stress level prediction model for mental health.

## Abbreviations

BMI: Body mass index
BUN: Blood urea nitrogen
Cr: Creatinine
HR: Heart rate
SLE: Stressful life event
